# Eriodictyol Suppresses *Porphyromonas gingivalis*-Induced Reactive Oxygen Species Production by Gingival Keratinocytes and the Inflammatory Response of Macrophages

**DOI:** 10.3389/froh.2022.847914

**Published:** 2022-02-28

**Authors:** Patricia Milagros Maquera-Huacho, Denise Palomari Spolidorio, John A. Manthey, Daniel Grenier

**Affiliations:** ^1^Oral Ecology Research Group, Faculty of Dentistry, Université Laval, Quebec City, QC, Canada; ^2^Department of Physiology and Pathology, School of Dentistry, São Paulo State University (Unesp), Araraquara, Brazil; ^3^U.S. Horticultural Research Laboratory, Agricultural Research Service, U.S. Department of Agriculture (USDA), Fort Pierce, FL, United States

**Keywords:** eriodictyol, macrophage, keratinocyte, *Porphyromonas gingivalis*, periodontal disease

## Abstract

*Porphyromonas gingivalis* is a key pathogen of periodontitis, an inflammatory disease that affects the tooth-supporting tissues. The aim of the present study was to investigate the effects of the flavanone eriodictyol on *P. gingivalis*-induced reactive oxygen species (ROS) production by gingival keratinocytes and the inflammatory response of macrophages. *Porphyromonas gingivalis* and H_2_O_2_ acted synergistically to induce ROS production by keratinocytes. The presence of eriodictyol significantly attenuated ROS production in a dose-dependent manner. We used a macrophage model to show that eriodictyol decreases the secretion of IL-1β, IL-6, IL-8, and TNF-α induced by *P. gingivalis*. Evidence has been brought that this anti-inflammatory property of eriodictyol may be related to its ability to prevent the activation of the NF-κB signaling pathway by *P. gingivalis*. This periodontal pathogen was also found to be a potent inducer of matrix metalloproteinase (MMP) production by macrophages, including MMP-2, MMP-8, and MMP-9. Eriodictyol dose-dependently inhibited the production of all three MMPs. Lastly, eriodictyol inhibited the catalytic activity of both MMP-9 and *P. gingivalis* collagenase. In conclusion, eriodictyol may be a potential therapeutic agent for preventing and/or treating periodontal disease due to its antioxidant, anti-inflammatory, and anti-proteinase properties.

## Introduction

Periodontal disease is a current public health problem worldwide that alters the quality of life of patients. More specifically, periodontitis, a destructive form of periodontal disease, affects nearly 10% of the USA adult population [1]. Periodontitis is defined as an inflammatory disorder of the periodontium triggered by specific anaerobic Gram-negative bacteria, called periodontal pathogens, that colonize the subgingival areas [2, 3]. If left untreated, periodontitis can lead to the destruction of tooth-supporting connective tissue and alveolar bone, leading to tooth loss. In addition to the damage caused in the oral cavity, the etiological factors of periodontitis have additional impacts on the whole human body. Associations between periodontitis and cardiovascular diseases, type 2 diabetes, neurodegenerative diseases, and adverse pregnancy outcomes have been reported by numerous studies [4–6].

In a healthy periodontium, the immune response of mucosal and infiltrating immune cells triggered by periodontal pathogens in the dental biofilm is moderate and protective [7, 8]. More specifically, macrophages and monocytes play a key role in host defenses against periodontal infections and contribute to the initiation of an adaptive immune response to periodontal pathogens such as *P. gingivalis* [9, 10]. However, if periodontal pathogens proliferate and increase in number, a state of dysbiosis develops in the subgingival sites, inducing an exaggerated inflammatory response, which leads to the secretion of cytokines and matrix metalloproteinases (MMPs) that sustain the chronic inflammatory reaction and modulate connective tissue and alveolar bone destruction [11]. The NF-κB signaling pathway, which consists of the NF-κB p50/p65 heterodimer and is inhibited by the suppressive protein inhibitor kappa B (IκB), plays a central role inflammatory mediator secretion [2, 7, 8]. Moreover, reactive oxygen species (ROS) production by resident and inflammatory cells of the periodontium is drastically increased during periodontitis and antioxidant host defense system cannot balance this process resulting in an oxidative stress condition and tissue damage [12].

*Porphyromonas gingivalis*, an anaerobic Gram-negative bacterium, is a key pathogen in chronic periodontitis [13, 14]. The presence of this bacterium in subgingival biofilm is associated with gingival bleeding, an increase in the depth of periodontal pockets, and an intensification of bone resorption [14]. Owing to the contribution of its numerous virulence factors [14–17], *P. gingivalis* can invade the gingival mucosa where it can elicit an inflammatory response following macrophage infiltration [18, 19].

**Figure 1:**
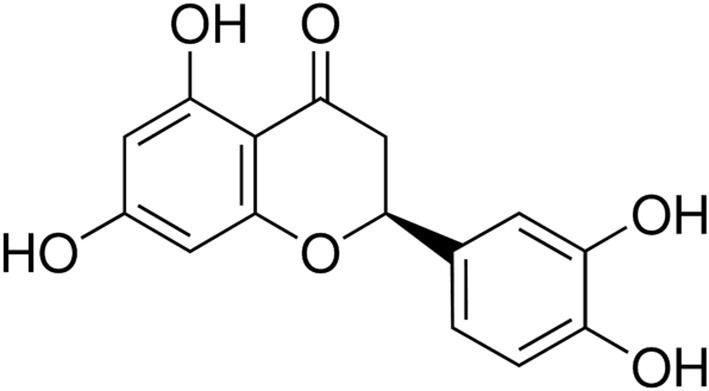
Molecular structure of eriodictyol.

Natural molecules isolated from plants may be promising alternatives to synthetic chemicals for the management of periodontal disease [20, 21]. Eriodictyol {Figure 1; [(S)-2-(3,4-dihydroxyphenyl)-5,7-dihydroxy-2,3-dihydrochromen-4-one]} is a flavonoid belonging to the flavanone subclass and is mainly found in the traditional medicinal plant yerba santa (*Eriodictyon californicum*) as well as in citrus fruits and vegetables [22, 23]. Studies carried out over the past decade have shown that eriodictyol exibits a number of therapeutic properties, including antioxidant and anti-inflammatory effects [22, 23]. The therapeutic properties of eriodictyol appear to depend on its capacity to modulate several cell signaling pathways [22, 23]. Although a recent study showed that food supplementation with eriodictyol could prevent periodontal disease in a mouse model [24], to the best of our knowledge, there are no reports in the literature that have addressed the exact underlying mechanisms of the potential beneficial effect of eriodictyol on periodontitis. In the present study, we investigated the effects of eriodictyol on *P. gingivalis*-induced ROS production by gingival keratinocytes and on the inflammatory response of macrophages.

## Materials and Methods

### Eriodictyol

Eriodictyol {Figure 1; [(S)-2-(3,4-dihydroxyphenyl)-5,7-dihydroxy-2,3-dihydrochromen-4-one]} was prepared at the USDA-ARS Horticultural Research Laboratory (Fort Pierce, Florida, USA). A stock solution was prepared in dimethylsulfoxide (DMSO) at a concentration of 1 M and was kept at 4°C in the dark. The final concentration of DMSO in the assays described below was ≤ 0.1% and had no effects on gingival keratinocytes and macrophages.

### Bacteria and Growth Conditions

*Porphyromonas gingivalis* ATCC 33277 was cultivated in an anaerobic chamber (80% N_2_, 10% CO_2_, 10% H_2_) at 37°C in Todd-Hewitt broth (THB; Becton Dickinson and Company, Sparks, MD, USA) containing 0.001% (w/v) hemin and 0.0001% (w/v) vitamin K.

### Gingival Keratinocytes and Reactive Oxygen Species Production

The previously characterized B11 human gingival keratinocyte cell line [25] was cultivated in keratinocyte serum-free medium (K-SFM; Life Technologies Inc., Burlington, ON, Canada) supplemented with 50 μg/mL of bovine pituitary extract, 5 ng/mL of human epidermal growth factor, 100 μg/mL of penicillin G-streptomycin, and 0.5 μg/mL of amphotericin B. Gingival keratinocytes (3 × 10^5^ cells/well) were seeded in black wall, clear flat bottom 96-well microplates (Greiner Bio-One North America, Monroe, NC, USA) and were incubated overnight at 37°C in a 5% CO_2_ atmosphere. ROS production was measured using a fluorometric assay to monitor the oxidation of 2′,7′-dichlorofluorescein-diacetate (DCF-DA; Sigma-Aldrich Canada Co., Oakville, ON, Canada) into a fluorescent compound. DCF-DA was freshly prepared in DMSO at a concentration of 40 mM. The cells were washed with Hank's balanced salt solution (HBSS; HyClone Laboratories, Logan, UT, USA) and were incubated for a further 30 min in the presence of 100 μM DCF-DA in HBSS. Excess DCF-DA was discarded, and the keratinocytes were washed with HBSS. The cells were then treated with *P. gingivalis* cells at a multiplicity of infection (MOI) of 100 either in the presence or absence of hydrogen peroxide (H_2_O_2_; 1 mM) and in the absence or presence of eriodictyol (40, 80, or 160 μM) in HBSS. H_2_O_2_ was used as an inducer of oxidative stress [26, 27]. The fluorescence emission indicating ROS production was then recorded every 20 min for 3 h at 37°C using a Synergy 2 microplate reader (BioTek Instruments, Winooski, VT, USA) with a 485 nm excitation filter and a 528 nm emission filter. Assays were carried out in triplicate in three independent experiments, and the means ± standard deviations were calculated.

### Adherence of *P. gingivalis* to Gingival Keratinocytes

To assess the effect of eriodictyol on the adhesion of *P. gingivalis* to gingival keratinocytes (B11 cell line), the bacteria were first labeled with fluorescein isothiocyanate (FITC). Briefly, a 24-h culture (10 mL) was harvested by centrifugation (9,000 × g for 5 min), washed once in 10 mm phosphate-buffered saline (PBS; pH 7.2), and suspended in 0.5 m sodium bicarbonate buffer (pH 8) containing 0.03 mg/mL of FITC. The bacterial suspension was incubated in the dark at 37°C for 30 min with constant shaking. The bacteria were then washed three times by centrifugation (9,000 × g for 5 min) and were suspended in 10 mL of K-SFM. Gingival keratinocytes cultured as described above were seeded at 1 × 10^6^ cells/mL in sterile black wall, clear flat bottom microplates (Greiner Bio-One North America Inc.) and were incubated overnight at 37°C in a 5% CO_2_ atmosphere. The medium was then removed by aspiration, and the confluent cell monolayers were pre-incubated with eriodictyol (40, 80, or 160 μM; in K-SFM) for 30 min at 37°C in a 5% CO_2_ atmosphere. FITC-labeled *P. gingivalis* was added at an MOI of 1,000, and the monolayers were incubated for a further 4 h at 37°C in a 5% CO_2_ atmosphere. Un-adhered bacteria were then removed, and the wells were washed twice with PBS. Relative fluorescence units (RFU) corresponding to the level of adhered bacteria were determined using a Synergy 2 microplate reader (BioTek Instruments) at an excitation wavelength of 495 nm and an emission wavelength of 525 nm. Wells with no *P. gingivalis* were used as controls to measure basal auto-fluorescence. Control wells without eriodictyol were used to determine 100% adherence values. Assays were performed in triplicate in three independent experiments, and the means ± standard deviations were calculated.

### Cytokine and MMP Secretion by *P. gingivalis*-Stimulated Macrophages

U937 human monocytes (CRL-1593.2; American Type Culture Collection, Manassas, VA, USA) were cultivated in Roswell Park Memorial Institute 1640 medium (RPMI; Life Technologies Inc.) supplemented with 10% heat-inactivated fetal bovine serum (FBS) and 100 μg/mL of penicillin G/streptomycin at 37°C in a 5% CO_2_ atmosphere. Prior to stimulation with *P. gingivalis*, the monocytes (1 × 10^6^ cells/mL) were differentiated into adherent macrophage-like cells by incubating them in RPMI-10% FBS containing 100 ng/mL of phorbol myristic acid (PMA; Sigma-Aldrich Canada Co.) for 48 h in 12-well microplates. After PMA treatment, the adherent macrophage-like cells were washed to remove remaining PMA and non-adherent cells. The differentiated cells were further incubated in RPMI-10% FBS without PMA for 24 h. Then, the adherent macrophage-like cells were maintained in RPMI-1% FBS and incubated overnight at 37°C in a 5% CO_2_ atmosphere. The macrophage-like cells were pre-treated for 2 h with eriodictyol (80, 160, or 320 μM) in RPMI-1% FBS prior to stimulating them with *P. gingivalis* cells at an MOI of 100. After a 24-h incubation at 37°C in a 5% CO_2_ atmosphere, the culture medium supernatants were collected and were kept at −20 °C until used. To exclude any effects resulting from a loss of cell viability, the cytotoxicity of eryodictyol at the concentrations used was assessed with an MTT (3-[4,5-diethylthiazol-2-yl]-2,5diphenyltetrazolium bromide) colorimetric assay (Roche Diagnostics, Laval, QC, Canada) performed according to the manufacturer's protocol. Cells incubated in culture medium in the absence of eriodictyol and bacteria were used as controls. Enzyme-linked immunosorbent assay (ELISA) kits (R&D Systems, Minneapolis, MN, USA; Invitrogen, Thermo Fisher Scientific Inc., Waltham, MA, USA) were used to determine IL-1β, IL-6, IL-8, TNF-α, MMP-2, MMP-8, and MMP-9 concentrations according to the manufacturers' protocols. Assays were performed in triplicate in three independent experiments, and the means ± standard deviations were calculated.

### NF-κB Activation in *P. gingivalis*-Stimulated Monocytes

The human monoblastic leukemia cell line U937 3xκ B-LUC, a subclone of the U937 cell line stably transfected with a luciferase gene linked to a promoter of three NF-κB-binding sites [28], was used to investigate the effect of eriodictyol on *P. gingivalis*-induced activation of the NF-κB signaling pathway. These cells were cultivated in RPMI-10% FBS, 100 μg/mL of penicillin G/streptomycin, and 75 μg/mL of hygromycin B at 37°C in a humidified incubator with a 5% CO_2_ atmosphere. The U937 3xκB-LUC monocytes were seeded (10^5^ cells/well) in the wells of black wall, black bottom, 96-well microplates (Greiner Bio-One North America) and were pre-incubated with eriodictyol (80, 160, or 320 μM) for 30 min. The monocytes were then stimulated with *P. gingivalis* at an MOI of 100 for 6 h. Wells containing monocytes without bacteria or eriodictyol were used as controls. Bright-Glo reagent (Promega Corporation, Durham, NC, USA) was used according to the manufacturer's protocol to measure luciferase activity and determine NF-κB activation. Luminescence was monitored using a Synergy 2 microplate reader. Assays were carried out in triplicate in three independent experiments, and the means ± standard deviations were calculated.

### Matrix Metalloproteinase-9 Activity

The effect of eriodictyol (80, 160, and 320 μM) on MMP activity was assessed using an MMP-9 Inhibitor Screening Fluorometric Assay Kit (Abcam Inc., Toronto, ON, Canada) according to the manufacturer's protocol. MMP-9 activity was monitored by recording fluorescence with a 328 nm excitation filter and a 420 nm emission filter for 20 min. N-isobutyl-N-(4-methoxyphenylsulfonyl)-glycylhydroxamic acid (NNGH; 1 mM) was used as a control inhibitor of MMP-9 activity. Assays were performed in triplicate in three independent experiments, and the means ± standard deviations were calculated.

### *P. gingivalis* Collagenase Activity

To determine the effect of eriodictyol on the collagenase activity of *P. gingivalis*, a 48-h culture was centrifuged at 10,000 × g for 10 min. The culture supernatant was incubated at 37°C for 3 h with eriodictyol (80, 160, or 320 μM) and 100 μg/mL of fluorogenic DQ substrate collagen (Molecular Probes, Eugene, OR, USA). The fluorescence corresponding to collagen degradation was measured every 30 min using a Synergy 2 microplate reader with a 495 nm excitation filter and a 525 emission filter. Leupeptin (1 μM) was used as a positive inhibitor control. Assays were performed in triplicate in three independent experiments, and the means ± standard deviations were calculated.

### Statistical Analysis

A one-way ANOVA with a *post-hoc* Bonferroni multiple comparison test was used to analyze the data. Results were considered statistically significant at *p* < 0.05, *p* < 0.01, or *p* < 0.001.

## Results

### Effect of Eriodictyol on ROS Production by Gingival Keratinocytes

**Figure 2:**
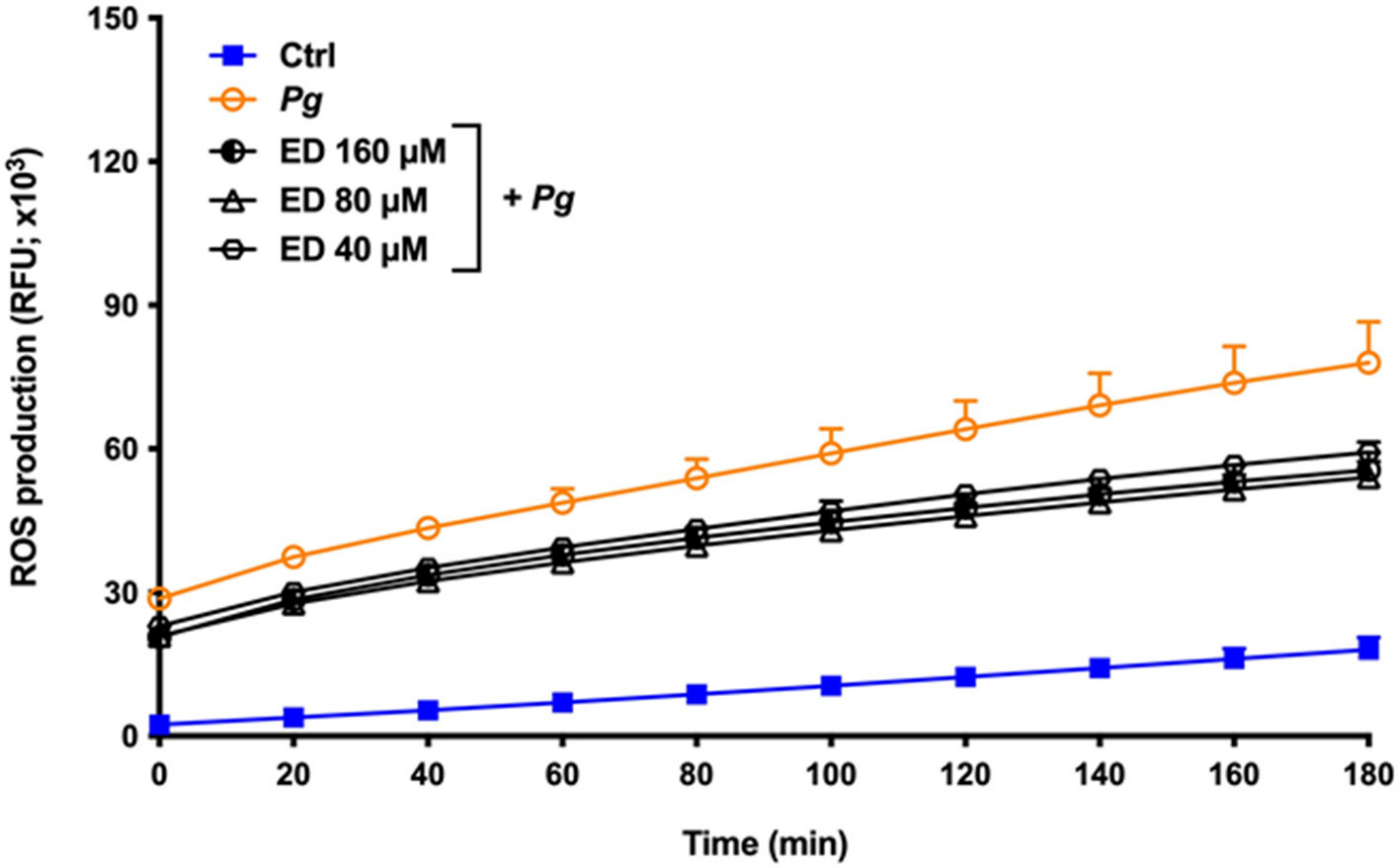
Time- and dose-dependent effects of eriodyctiol on ROS production by gingival keratinocytes treated with *P. gingivalis* (MOI = 100). Results are expressed as the means ± SD of triplicate assays from three independent experiments. All values obtained in the presence of eriodictyol are significantly different from *P. gingivalis-*infected cells not treated with eriodictyol (*p* < 0.05).

**Figure 3:**
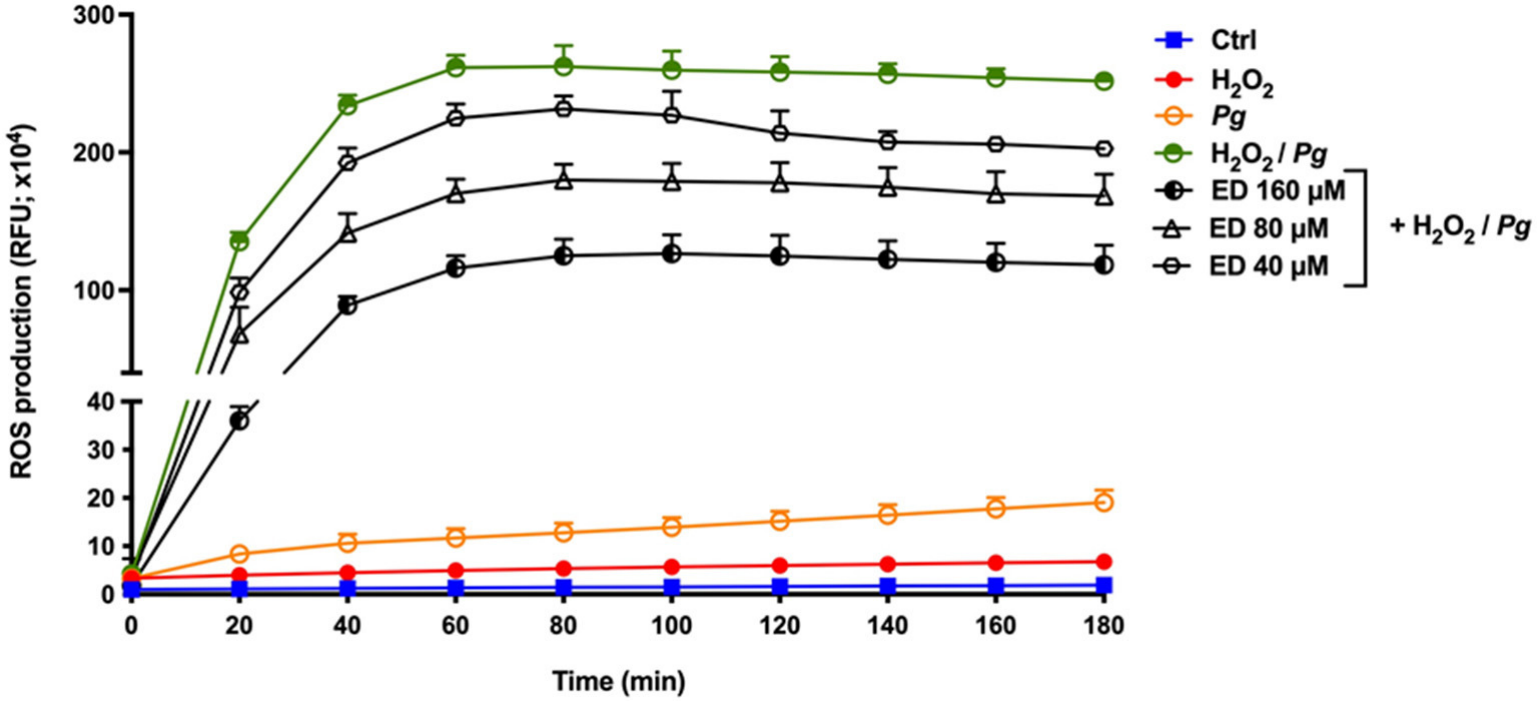
Time- and dose-dependent effects of eriodictyol on ROS production by gingival keratinocytes stimulated with *P. gingivalis* + H_2_O_2_. Results are expressed as the means ± SD of triplicate assays from three independent experiments. All values obtained in the presence of eriodictyol are significantly different from keratinocytes stimulated with *P. gingivalis* + H_2_O_2_ in the absence of eriodictyol (*p* < 0.05).

The production of ROS by gingival keratinocytes may cause deleterious effects on the oral epithelium. It was first shown that *P. gingivalis* (MOI = 1,000) time-dependently increased the production of ROS by gingival keratinocytes. Following a 3-h contact, we observed a 4.3-fold increase in ROS compared to untreated control cells (Figure 2). However, in the presence of eriodictyol, at all the concentrations tested (40, 80, and 160 μM), *P. gingivalis*-induced ROS production was significantly attenuated, with a 24.0–30.6% reduction after the 3-h contact (Figure 2). The ability of eriodictyol to impair ROS production by gingival keratinocytes treated with *P. gingivalis* combined with H_2_O_2_, a well-known inducer of oxidative stress [26, 27], was also investigated. As reported in Figure 3, *P. gingivalis* and H_2_O_2_ acted synergistically to induce ROS production by gingival keratinocytes. More specifically, following a 3-h contact, the *P. gingivalis* + H_2_O_2_ combination induced a 19.5-fold higher production of ROS compared to the amount of ROS produced by cells treated with *P. gingivalis* or H_2_O_2_ alone. Eriodictyol attenuated ROS production, with a 160 μM concentration causing a 52.9% inhibition after a 3-h contact.

### Effect of Eriodictyol on Adhesion of *P. gingivalis* to Gingival Keratinocytes

**Figure 4:**
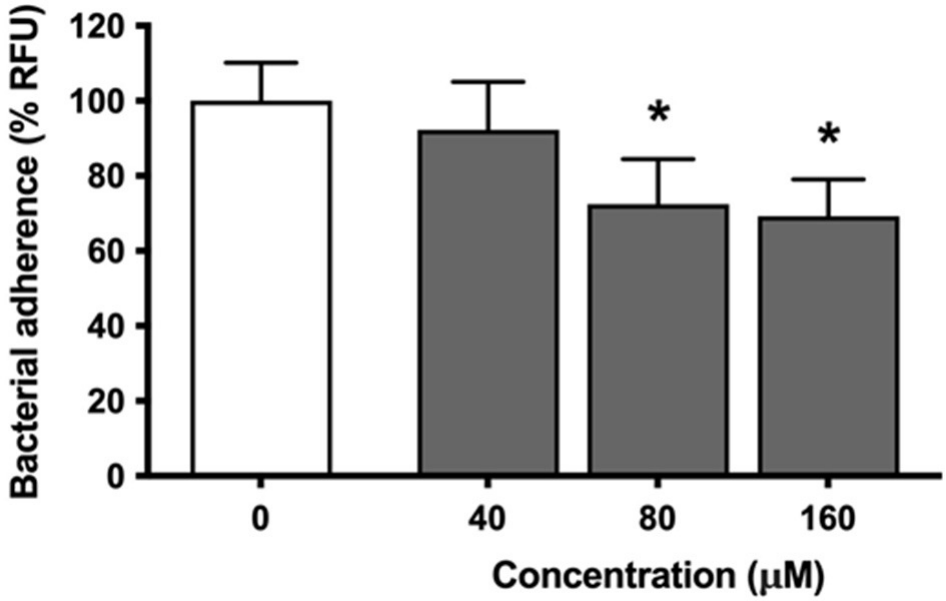
Dose-dependent effect of eriodictyol on the adherence of *P. gingivalis* to gingival keratinocytes. Results are expressed as the means ± SD of triplicate assays from three independent experiments. ^*^Values are significantly different from those of the adherence assay performed in the absence of eriodictyol (*p* < 0.05).

Analyses were then conducted to determine the ability of eriodictyol to inhibit the adhesion of *P. gingivalis* to gingival keratinocytes (B11 cell line) as a potential mechanism involved in the inhibition of *P. gingivalis*-induced ROS production. Eriodictyol dose-dependently reduced the adherence of FITC-labeled *P. gingivalis* to gingival keratinocytes (Figure 4). More specifically, in the presence of 160 μM eriodictyol, adherence was inhibited by 30.8%.

### Effect of Eriodictyol on Cytokine and MMP Production by Macrophages

**Figure 5:**
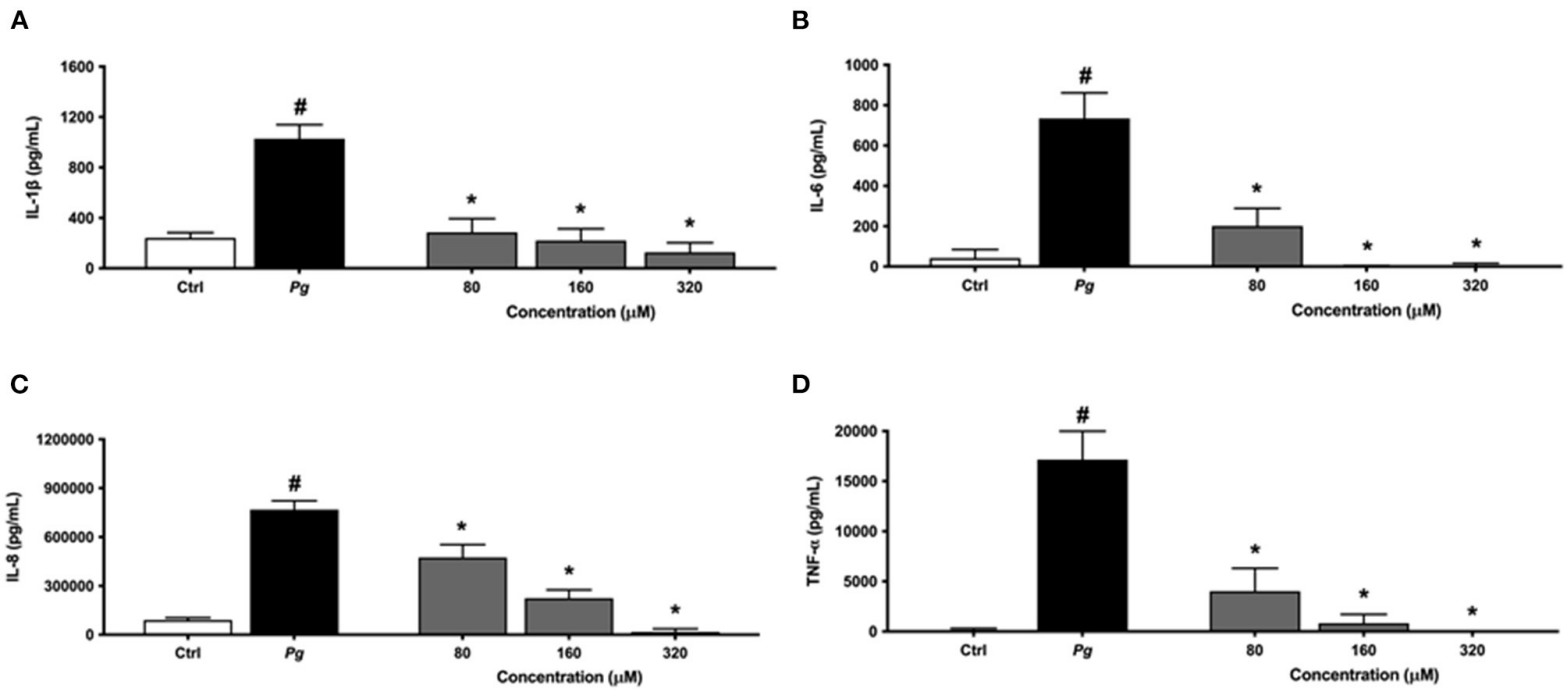
Dose-dependent effect of eriodictyol on the secretion of IL-1β **(A)**, IL-6 **(B)**, IL-8 **(C)**, and TNF-α **(D)** by macrophages stimulated with *P. gingivalis* (MOI = 100) for 24 h. Results are expressed as the means ± SD of triplicate assays for three independent experiments. ^#^Significant increase (*p* < 0.001) compared to cells not stimulated with *P. gingivalis*. ^*^Significant decrease (*p* < 0.001) compared to *P. gingivalis*-stimulated cells.

To investigate the anti-inflammatory effect of eriodictyol, macrophages were pretreated for 2 h with various concentrations (80, 160, or 320 μM) of this flavanone before being challenged with *P. gingivalis* cells at an MOI of 100. To exclude the possibility that the reduction in levels may have resulted from cell toxicity, we evaluated the viability of macrophages that had been treated for 24 h with eriodictyol. No obvious cytotoxic effects were detected with eriodictyol at a final concentration of 320 μM. Cell viability was ≥92% compared with the untreated control (data not shown). The secretion of IL-6, IL-8, TNF-α, and IL-1β increased significantly when the macrophages were stimulated with *P. gingivalis* compared to untreated cells (Figure 5). More specifically, the secretion of IL-6, IL-8, TNF-α, and IL-1β increased 17.4-, 8.5-, 138.5-, and 4.2-fold, respectively. Eriodictyol, at all the concentrations tested, significantly decreased the *P. gingivalis*-induced secretion of the four cytokines. In the presence of 160 μM eriodictyol, the secretion of IL-6, IL-8, TNF-α, and IL-1β decreased by 99.9, 70.8, 95.2, and 78.5%, respectively.

**Figure 6:**
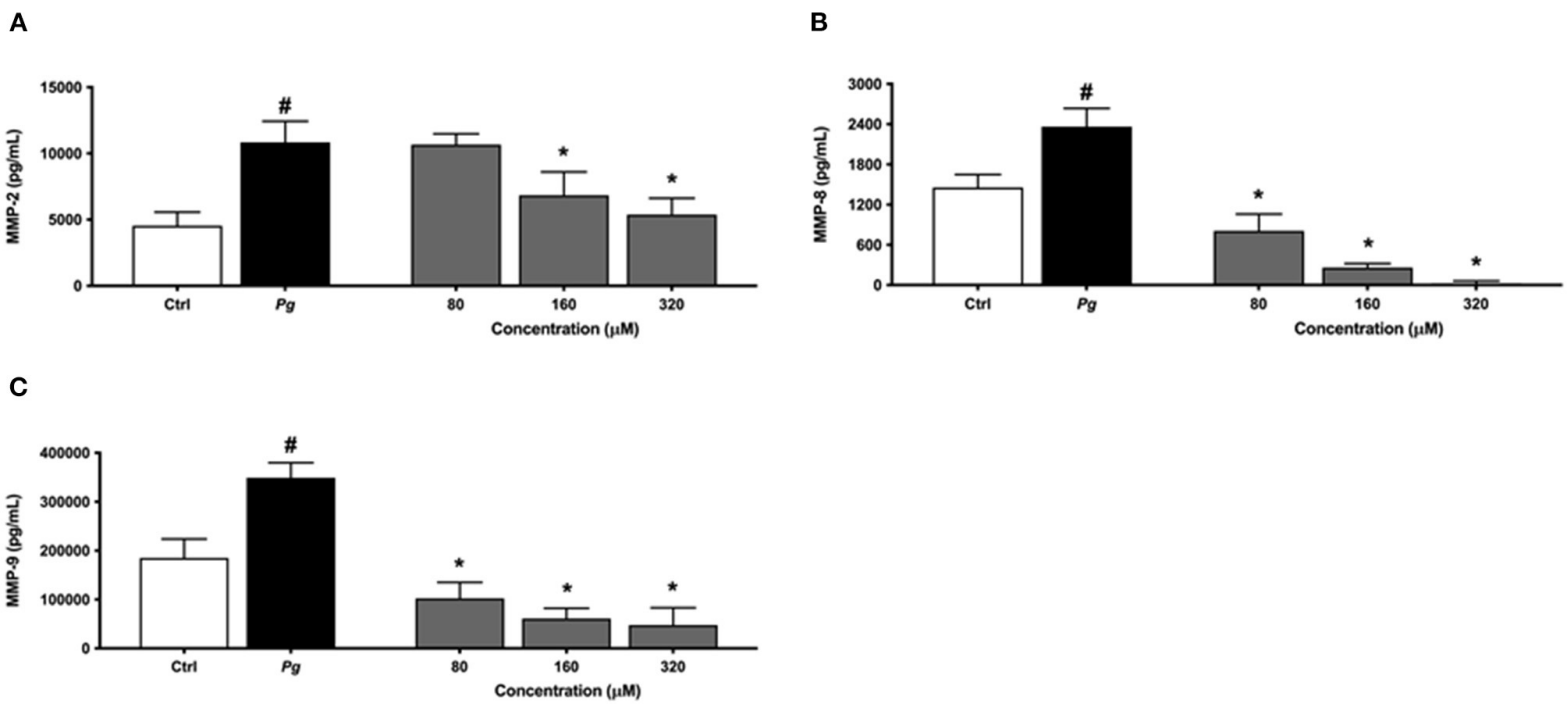
Dose-dependent effect of eriodictyol on the secretion of MMP-2 **(A)**, MMP-8 **(B)**, and MMP-9 **(C)** by macrophages stimulated with *P. gingivalis* (MOI = 100) for 24 h. Results are expressed as the means ± SD of triplicate assays for three independent experiments. ^#^Significant increase (*p* < 0.001) compared to cells not stimulated with *P. gingivalis*. ^*^Significant decrease (*p* < 0.001) compared to *P. gingivalis*-stimulated cells.

The same protocol was used to assess the effect of eriodictyol on MMP production by *P. gingivalis*-treated macrophages. The secretion of MMP-2, MMP-8, and MMP-9 increased significantly when the macrophages were challenged with *P. gingivalis* compared with untreated cells (Figure 6). Eriodictyol dose-dependently reduced the production of all three MMPs. At a concentration of 160 μM, eriodictyol reduced the *P. gingivalis*-induced production of MMP-2, MMP-8, and MMP-9 by 36.9, 99.9, and 82.5%, respectively.

### Effect of Eriodictyol on NF-κB Activation in a Monocyte Model

**Figure 7:**
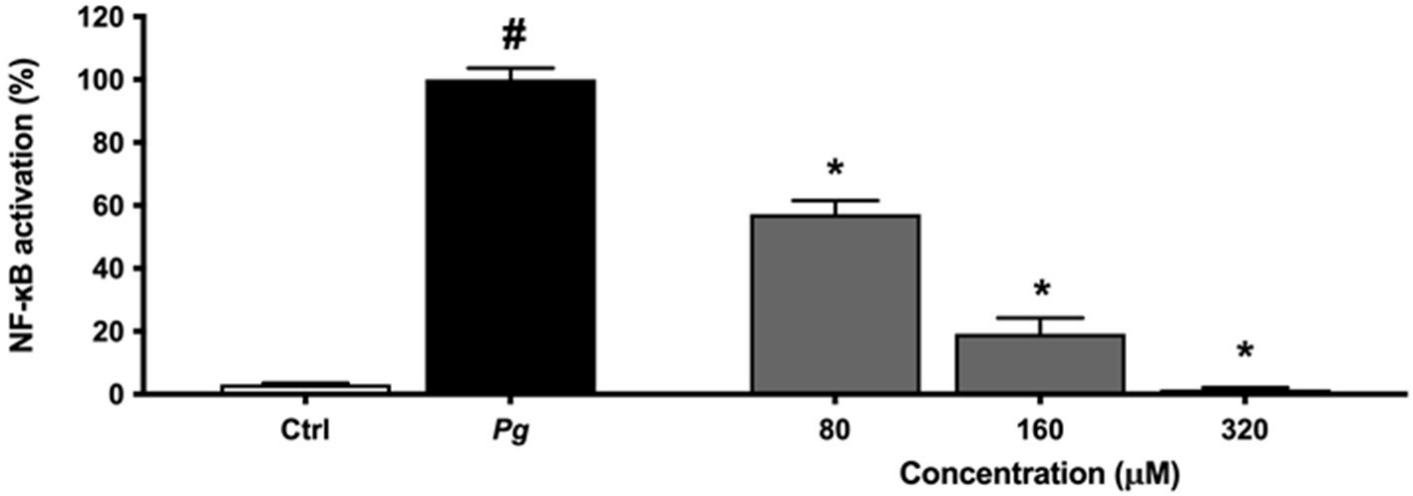
Dose-dependent effect of eriodictyol on *P. gingivalis* (MOI = 100)-induced NF-κB activation. A value of 100% was assigned to the activation obtained with *P. gingivalis* in the absence of test compounds. Results are expressed as the means ± SD of triplicate assays for three independent experiments. ^#^Significant increase (*p* < 0.01) compared to cells not stimulated with *P. gingivalis*. ^*^Significant decrease (*p* < 0.01) compared to *P. gingivalis*-stimulated cells.

An alternative model was used to assess the anti-inflammatory effect of eriodictyol. Since NF-κB is a transcription factor implicated in inflammatory processes leading to the production of cytokines and MMPs, we evaluated the effect of eriodictyol on NF-κB activation using the U937 3xκ B-LUC monocytic cell line. As shown in Figure 7, eriodictyol dose-dependently reduced *P. gingivalis*-modulated NF-κB activation. At a concentration of 160 μM, eriodictyol reduced NF-κB activation by 80.7%.

### Effect of Eriodictyol on the Catalytic Activity of MMP and *P. gingivalis* Collagenase

**Figure 8:**
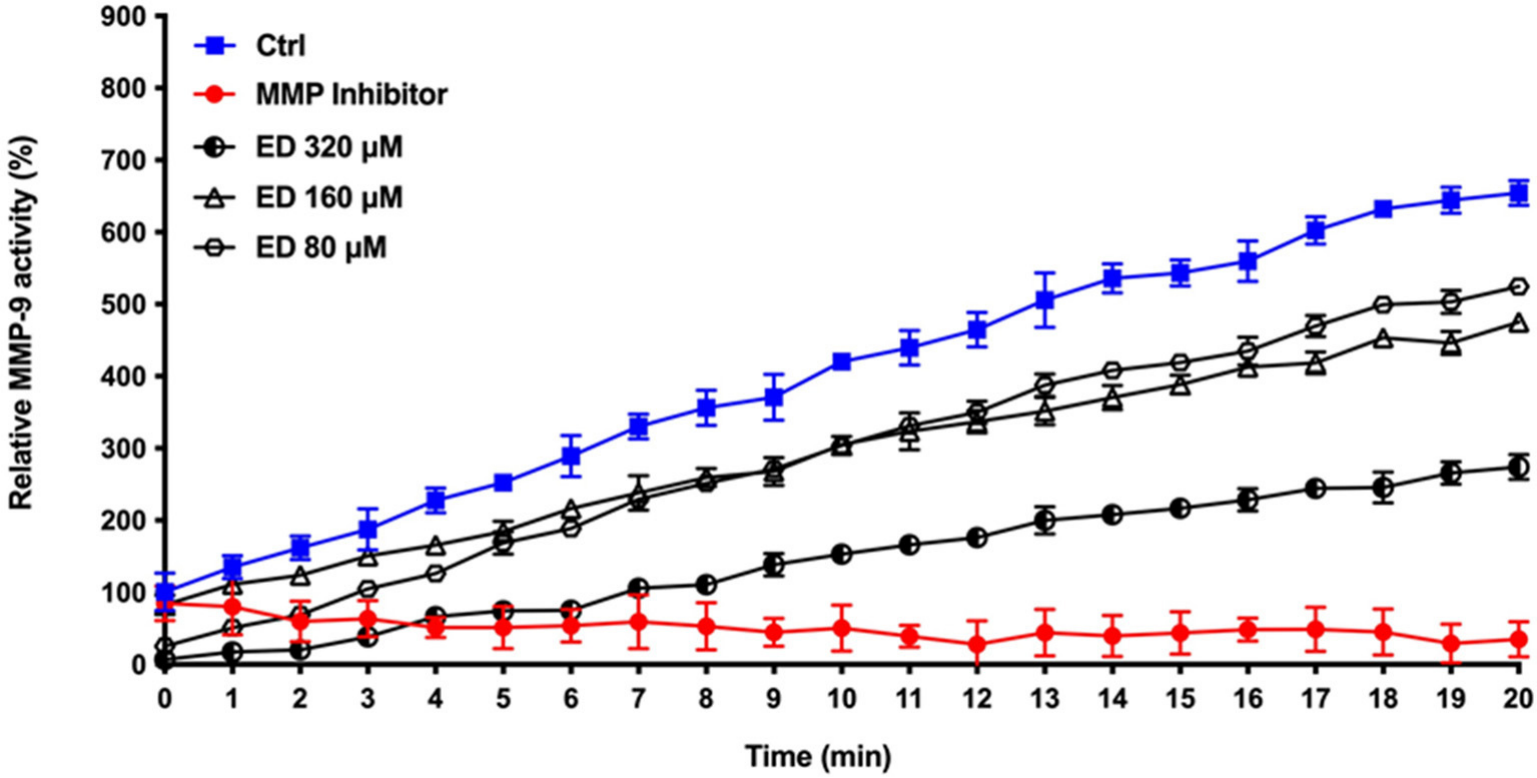
Dose- and time-dependent effects of eriodictyol on the activity of MMP-9. Results are expressed as the means ± SD of triplicate assays from three independent experiments. All values obtained in the presence of eriodictyol are significantly different from the assay performed in the absence of eriodictyol (*p* < 0.05).

Eriodictyol was further studied for its ability to inhibit MMP activity. We used MMP-9 as a model and showed that eriodictyol inhibits the catalytic activity of MMP-9 in a dose- and time-dependent manner (Figure 8). After the 20-min incubation time, eriodictyol at 80, 160, and 320 μM inhibited MMP-9 activity by 19.9, 27.4, and 58.0%, respectively.

**Figure 9:**
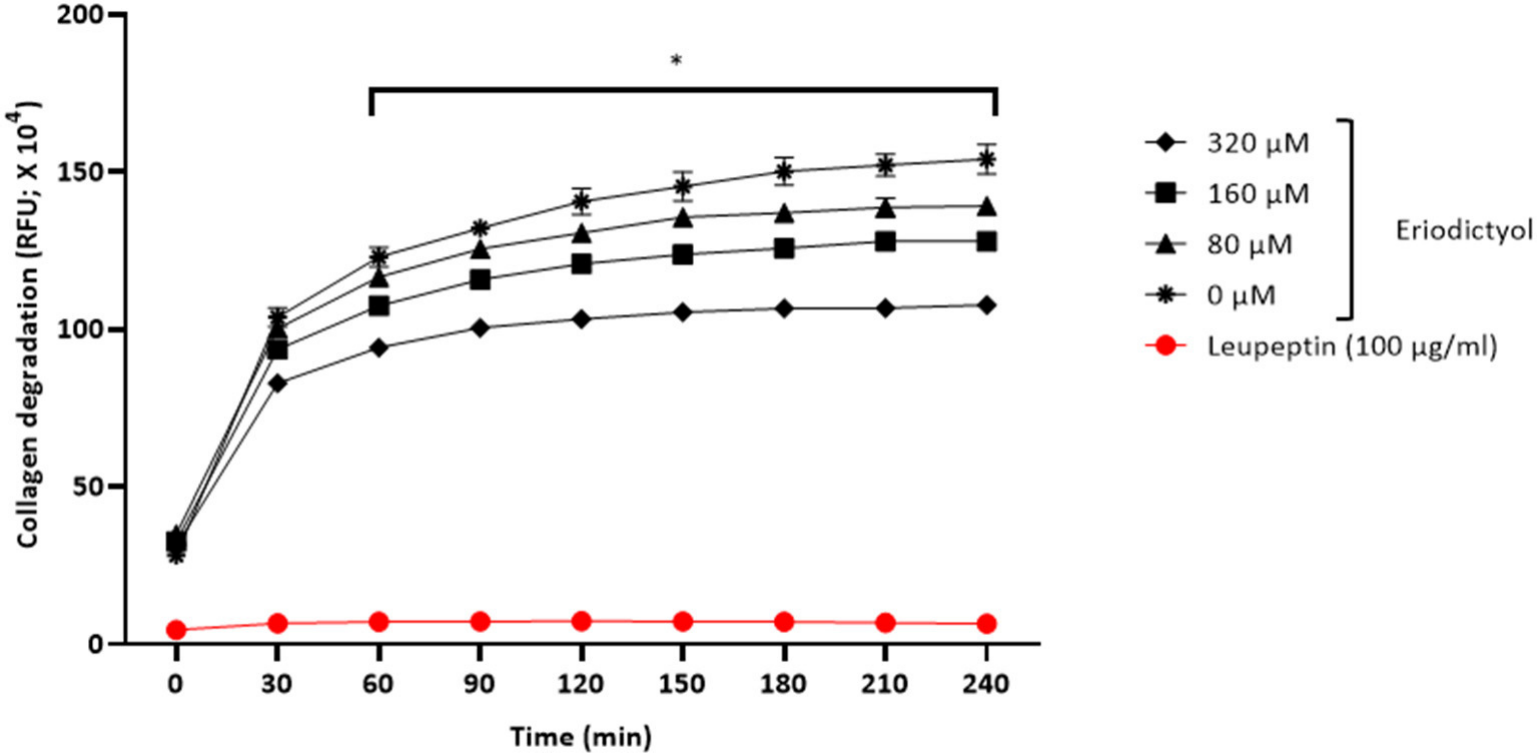
Dose- and time-dependent effects of eriodictyol on the degradation of collagen by *P. gingivalis*. Results are expressed as the means ± SD of triplicate assays from three independent experiments. ^*^Values are significantly different from the assay performed in the absence of eriodictyol (*p* < 0.05).

Given that *P. gingivalis* may act in concert with MMPs to cause the destruction of periodontal connective tissue, we assessed the effect of eriodictyol on the collagenase activity of this periodontal pathogen. We showed that eriodictyol inhibits the degradation of type I collagen by *P. gingivalis* in a dose- and time-dependent manner. After a 4-h incubation, the lowest concentration (80 μM) of eriodictyol inhibited collagen degradation by 9.6% while the highest concentration (320 μM) inhibited collagen degradation by 30.1% (Figure 9).

## Discussion

Periodontitis is a chronic inflammatory disease associated with an overproduction of cytokines and MMPs, which mediate connective tissue destruction and bone resorption. It results from a dysbiosis of the commensal oral microbiota, resulting in the overgrowth of a specific group of Gram-negative bacteria, including *P. gingivalis* [11, 29]. Oxidative stress and the inflammatory response mediated by periodontal pathogens are major contributors to periodontitis [2, 7, 30, 31]. Consequently, host modulation therapies involving antioxidant and anti-inflammatory compounds are a promising avenue for the treatment of this disease [32]. The search for novel antioxidant and anti-inflammatory agents is an active area of research as traditional therapies are often associated with undesirable side effects. In this regard, plant polyphenols are interesting bioactive molecules given their ability to act on multiple inflammatory targets [20, 21]. The aim of the present study was to investigate the effects of the flavanone eriodictyol on *P. gingivalis*-induced ROS production by gingival keratinocytes and on the inflammatory response of macrophages.

Oxidative stress resulting from an imbalance between oxidative and anti-oxidative episodes has been associated with various inflammatory diseases, including periodontitis [30, 31, 33]. More specifically, elevated levels of ROS originating from oxidative stress have been detected in the saliva and gingival crevicular fluid of periodontitis subjects [34–36]. These excessive amounts of ROS may cause damage to DNA, proteins, and lipids, and may also induce an inflammatory response in resident and immune cells in diseased periodontal sites [37, 38]. Given that the oral mucosa is in close contact with periodontal pathogens, we used a gingival keratinocyte model to show that *P. gingivalis* cells induce the production of intracellular ROS. This agrees with the results of Wang et al. [39], who reported that stimulating gingival epithelial cells with *P. gingivalis* induced the production of ROS, which caused the phosphoactivation of JAK2 and led to the secretion of the pro-inflammatory cytokines IL-6 and IL-8. Interestingly, we showed that *P. gingivalis* and H_2_O_2_, when used together, act in synergy to produce significantly higher amounts of ROS. To the best of our knowledge, this is the first time that such a synergistic effect has been reported. The presence of eriodictyol during keratinocyte stimulation with *P. gingivalis* ± H_2_O_2_ significantly inhibited ROS production in a dose-dependent manner. The fact that eriodictyol was also shown to inhibit the adhesion of *P. gingivalis* to gingival keratinocytes may explain, at least in part, its ability to attenuate ROS production. The antioxidant activity of eriodictyol has been previously reported in studies using various cell models, including mesangial cells [40], macrophages [41], and microglial cells [42]. Given that a periodontal therapy that improves the status of oxidative stress has been shown to be beneficial [43], eriodictyol may be of interest for such a therapeutic approach.

Macrophages, which are key cells of the innate immune system, play an essential role in the overall inflammatory response. During the onset and progression of periodontitis, macrophages are found in high numbers in diseased periodontal sites [9, 10]. These leukocytes modulate the inflammation process by secreting a variety of chemical agents, including cytokines, that can amplify the inflammatory response [9, 10]. We showed that eriodictyol attenuates the production of IL-1β, IL-6, IL-8, and TNF-α by *P. gingivalis*-stimulated macrophages. Eriodictyol may thus be a promising molecule for modulating the inflammatory process that occurs during periodontitis. Studies aimed to investigate the ability of eriodictyol to up-regulate the secretion of anti-inflammatory cytokines such as IL-4 and IL-10 may further support the anti-inflammatory potential of eriodictyol.

NF-κB is considered the main mediator of the immune response and is a central pathway by which host cells respond to stress and bacterial challenges [44]. Consequently, NF-κB is a key target for anti-inflammatory compounds as inhibiting it may impair pro-inflammatory cytokines secretion [45]. We used the U937 3xκ BLUC cell line to show that *P. gingivalis* cells activate the NF-κB signaling pathway and that eriodictyol almost completely prevents this activation. It can be hypothesized that inhibition of NF-κB activation may be related to the ability of eriodictyol to prevent phosphorylation and subsequent degradation of IκB. Our results agree with the study of Lee [46], who reported that eriodictyol reduces mRNA expression and secretion of pro-inflammatory cytokines in a murine macrophage model. This anti-inflammatory property involves the blockage of NF-κB activation.

Macrophages, which make up an important part of the inflammatory infiltrate in active periodontal lesions, are major producers of MMPs when they are triggered by periodontal pathogens [10]. Given their action on a large array of extracellular matrix proteins such as laminin, fibronectin, and collagen, MMPs contribute to periodontal tissue destruction [47, 48]. In the present study, we showed that *P. gingivalis* cells act as a potent inducer of MMP production, including MMP-2,−8, and−9, by macrophages. Eriodictyol inhibited the production of all three MMPs, suggesting that the use of this flavanone may be a potentially effective strategy for the prevention and control of periodontitis.

Periodontal tissue destruction has been associated with the presence of high levels of active MMP-9 in gingival tissue, saliva, and gingival crevicular fluid [47–49]. Moreover, this MMP may be a practical marker for assessing the clinical severity of periodontitis [50]. We thus used MMP-9 as an MMP model to investigate the ability of eriodictyol to inhibit the catalytic activity of MMPs. We showed that eriodictyol markedly inhibits MMP-9 activity.

Eriodictyol, by inhibiting both the production and the activity of MMPs, offers a promising perspective for developing novel and innovative therapies for treating pathologic conditions characterized by excessive MMP activity such as in periodontitis. In addition to MMPs, the collagenase activity of *P*. *gingivalis* may also participate in periodontal tissue breakdown. Type I collagen makes up ~60% of the periodontal tissue volume. In the present study, we showed that eriodictyol reduces collagen degradation by *P*. *gingivalis*, suggesting that it may contribute to attenuating the tissue destruction process.

Given that periodontitis is a complex disease initiated by a polymicrobial biofilm colonizing the subgingival sites, a limitation of this study is the fact that a single periodontal pathogen grown in planktonic culture was used to stimulate gingival keratinocytes and macrophages. Nevertheless, eriodictyol, incorporated into mouthrinses, toothpastes, gels, or local drug delivery systems, may represent a promising therapeutic agent for the prevention and/or treatment of periodontal disease due to its antioxidant, anti-inflammatory, and anti-MMP properties.

## Data Availability Statement

The original contributions presented in the study are included in the article/supplementary material, further inquiries can be directed to the corresponding author.

## Author Contributions

DG, PMMH, and DPS conceived and designed the experiments. PMMH performed the experimental assays and the statistical analysis. JM analyzed and provided the test compound for the study. DG drafted the manuscript. DG, PMMH, JM, and DPS reviewed and edited the manuscript. All authors contributed to the article and approved the submitted version.

## Funding

PMMH was supported by a scholarship provided by São Paulo Research Foundation (FAPESP; Grant #2018/16540-8 and #2019/15343-7). This study was funded by the Laboratoire de Contrôle microbiologique of Université Laval (2021-12).

## Conflict of Interest

The authors declare that the research was conducted in the absence of any commercial or financial relationships that could be construed as a potential conflict of interest.

## Publisher's Note

All claims expressed in this article are solely those of the authors and do not necessarily represent those of their affiliated organizations, or those of the publisher, the editors and the reviewers. Any product that may be evaluated in this article, or claim that may be made by its manufacturer, is not guaranteed or endorsed by the publisher.
